# Mortality and Years of Life Lost due to Burn Injury Among Older Iranian People; a Cross-Sectional study

**DOI:** 10.22037/aaem.v10i1.1547

**Published:** 2022-04-27

**Authors:** Farideh Sadeghian, Sahar Saeedi Moghaddam, Zahra Ghodsi, Parinaz Mehdipour, Ali Ghanbari, Gerard O'Reilly, Nazila Rezaei, Sahar Mohammadi Fateh, Ali H. Mokdad, Vafa Rahimi-Movaghar

**Affiliations:** 1 Center for Health Related Social and Behavioral Sciences Research, Shahroud University of Medical Sciences, Shahroud, Iran.; 2 Sina Trauma and Surgery Research Center, Tehran University of Medical Sciences, Tehran, Iran.; 3 Non-Communicable Diseases Research Center, Endocrinology and Metabolism Population Sciences Institute, Tehran University of Medical Sciences, Tehran, Iran.; 4 Endocrinology and Metabolism Research Center, Endocrinology and Metabolism Clinical Sciences Institute, Tehran University of Medical Sciences, Tehran, Iran.; 5 Centre for Epidemiology and Biostatistics, Melbourne School of Population and Global Health, University of Melbourne, Melbourne, Australia.; 6 Department of Epidemiology and Preventive Medicine, School of Public Health and Preventive Medicine, Monash University, Melbourne, Australia.; 7 Institute for Health Metrics and Evaluation, University of Washington, Washington, USA.; 8 Brain and Spinal Cord Injury Research Center, Neuroscience Institute, Tehran University of Medical Sciences, Tehran, Iran.; 9 Department of Neurosurgery, Shariati Hospital, Tehran University of Medical Sciences, Tehran, Iran.

**Keywords:** Burns, Aged, Mortality, Wounds and Injuries, Years of Life Lost

## Abstract

**Introduction:**

The mortality of burn injury is a serious health problem among older people. The present study aimed to determine the epidemiological characteristics of burn mortality and Years of Life Lost (YLLs) among people aged ≥ 60.

**Methods:**

The National and Subnational Burden of Disease (NASBOD) study includes population-based cross-sectional data from the death registration system of Iran and those recorded by the cemeteries of Tehran and Esfahan were used in this study. Spatio-temporal and Gaussian process regression models were applied to estimate rates and trends of mortality and cause-specific mortality fractions. YLLs were calculated using Iranian life expectancy and the number of deaths.

**Results:**

The mortality rate for 1990 and 2015 was 17.4 and 4.5 per 100,000, respectively. From 1990 through 2015, the annual percentage of change in burn mortality rate was -6.1% in females and -4.4% in males. During 2015, there were 326 deaths following burns in people aged 60+ with 4586 person YLLs, and in 1990 there were 523 deaths with 4862 person-YLLs. The male-female ratio for 1990 and 2015 were 0.80 and 0.88, respectively. The age-standardized mortality rate was higher than 8.5 per 100,000 in border provinces in 2015. The provinces with better socioeconomic situations, such as Tehran, had a lower mortality rate than poor provinces, such as Sistan va Baluchistan.

**Conclusion:**

Although burn mortality in old people decreased in those 26 years, it is still high compared to high-income countries. Continued efforts to increase preventive measures and adequate access to quality care, especially in border provinces, is suggested.

## 1. Introduction

Burn injuries, with mortality of 265,000 per year, are a major public health matter worldwide (1). Although economic growth has decreased burn deaths in younger age- groups, mortality rates have increased among the < 65-year-old population globally (2). Burns are a serious injury among older people with an even greater burden of mortality (3, 4). A systematic review study indicated that the rate of death in the elderly over 75 years old was two times more than that of those under 65 years old (5). In the United States, from 1999 to 2015, residential fires caused 45,000 deaths, people ≥ 65 years of age were reliably at the highest risk (6, 7).

Analysis of the Canadian National Fire Information database indicated that mortality due to fire-related injuries was much more probable among older people than the overall population, so the relative risk (RR) of burn injuries in 65 to 79 and 80 ≤ years is 1.6 and 2.4 times more than the overall population, respectively (8). 

In a longitudinal study in Australia from 1980 to 2012, among older hospitalized people aged at least 45 years, 180 (3%) died in hospital. Of the 2498 (42%) patients who died after discharge, 1442 patients were aged≥ 65 years old (9). A Swedish study calculated remaining Life Expectancy (rLE) among 111 previous burn patients and 77 of them (69%) died before reaching the rLE, with a mean of 4.7 years of life lost (YLLs). The results showed that the long-term survival of aging patients with subsequent burns is shorter than that of a national control (10). 

Of the 819 unintentional burn injuries in a tertiary hospital in Nepal, from 2002 to 2013, 11% (88 patients) were aged more than 65 (11). Studies indicated a close relationship between injury mortality and economic progress, therefore age-specific fire mortality rates can differ greatly between countries (12). Most of the mortality resulting from fire-related burns occur in low- and middle-income countries (13). In Iran, burn injury is ranked second after traffic injuries in all trauma causes (14). Among burn patients in a hospital in Mashhad, a city in Iran, the burn mortality rate increased from 3.3% among patients less than 6 years of age to 51.2% in patients > 65 years (15). In a study performed in Tehran, Iran, from 2007 to 2014, the mean age of patients aged ≥ 60 years, who were admitted to the Burn Center with acute burns was 71.5 years (16). In a study of 1618 burn injury patients admitted to Sina Academic Burn Centre in Tabriz mortality rates were 3.18% in those aged less than 16 years, 7.98% in those aged 16- 44 years, 7.11% in the 44-64 years age group, and 12.04% in the >64 years age group(17). High-income countries (HICs) have had great progress in reducing rates of burn mortality, such that survival has become a predictable result even in patients with extensive burn injuries. While these improvements in outcome have occurred in all age groups, there have been relatively fewer improvements in survival of older adults compared to younger adults(3). Also, most of these preventive programs have been partly practical in low and middle-income countries (LMICs) (18, 19). 

According to WHO, it is expected that, the population aged≥60 will rise from 12% to 22%, globally, during 2015 - 2050. As a result of prolonged independent living of the elderly, burn injuries in the elderly population will be increasing due to impaired daily function but they are a medically challenging group for specialized burn care (20). In recent years, epidemiological studies have highlighted that old people still lag behind younger burn patients regarding death and other related results (21-23).

There is a dearth of research focusing on the burn mortality of the elderly in developing countries. Burn mortality in Iran is high (14) and the age trend of the people is likely to result in an increasing burden from burns in future decades in Iran. There has been a lack of research on burn mortality among the elderly in Iran (14). 

As the global health observatory states, the total amount of death for a specific reason is not a good measure for determination of its public heath importance, as the same weight is given to deaths at any age. YLL is a better measure in this regard as it considers both mortality frequency and the age of death(24).

Epidemiological studies must be performed in order to establish special information to be provided as the basis for prevention programs, for their design and implementation. 

To the best of our knowledge, this paper is the first one approaching the problem of burn injuries among older people in Iran, which may provide the basis for design and implementation of prevention plans, and consequently help decrease the rate of burn-related mortality among old people. Therefore, our hypothesis was to determine the mortality rate and YLLs of burn injuries among Iranian people aged 60 or older at national and provincial levels. 

## 2. Methods

***2.1*** ***.*** ***Study design and setting***

The National and Subnational Burden of Disease (NASBOD) study data was used in this study. This data estimated the burden due to 290 diseases and reported the burden due to 67 risk factors from 1990 to 2015 based on age groups and sex at national and provincial levels in Iran(25). Methodological details of the NASBOD study have been explicitly described previously (26-31). Methods of estimating death and YLL due to burn injury, to focus on elderly people, are briefly reviewed here.

After receiving the population-based cross-sectional data of the death registration system across all provinces of the country and the data recorded by the cemeteries of Tehran and Isfahan (Behesht-e-Zahra and Bagh-e-Razvan), an analytical team of statistical and epidemiology experts was formed. The physician team has worked with the analysis team to check the clinical validity of the data for statistical analysis. ICD-10 codes of X00-X19 were mapped to define death due to burn.

The Ethics Committee of Tehran University of Medical Sciences approved the study with the reference number of IR.TUMS.EMRI.REC.1396.00175.

***2.2*** ***.*** ***Statistical analysis***

Multiple imputations by the Amelia package in R statistical software were used to impute sex and age. Further, two-stage multiple imputation was used to impute the cause of death. Spatio-temporal and Gaussian process regression models were applied to estimate levels and trends of child and adult mortality rates (29-31). Mixed effect and Spatio-temporal models were applied to assess cause-specific mortality fraction. Cause-specific mortality was derived by applying cause-specific mortality fraction to all causes of mortality (27) . Total years of schooling, wealth index, and urbanization were measured as covariates. YLLs were calculated using several death causes and Iranian life expectancy obtained from a parallel study. All these estimates were provided based on age, sex, and year at national and sub-national levels. We report death and YLL in 5-year age intervals as well as in all ages (60+) and in an age-standardized manner. To compare estimates across provinces, the national population of Iran in 2015 was used as the standard population in the direct age-standardized approach. Annual percentage change (APC) was calculated as the exponential coefficient from a regression of the natural logarithm of the annual rate or number for each measure (death/YLLs). R statistical software (R Foundation for Statistical Computing; Vienna, Austria) and STATA (Stata Corp LP, TX, and the USA) software were used for statistical analyses.

## 3. Results:

A total of 523 patients (290 females and 233 males) aged 60+ years died as a result of burn injury in Iran in 1990 and this measure was 326 (173 females and 153 males) in 2015. The male/female ratio in 1990 and 2015 was 0.8 and 0.88, respectively.

**Figure 1:**
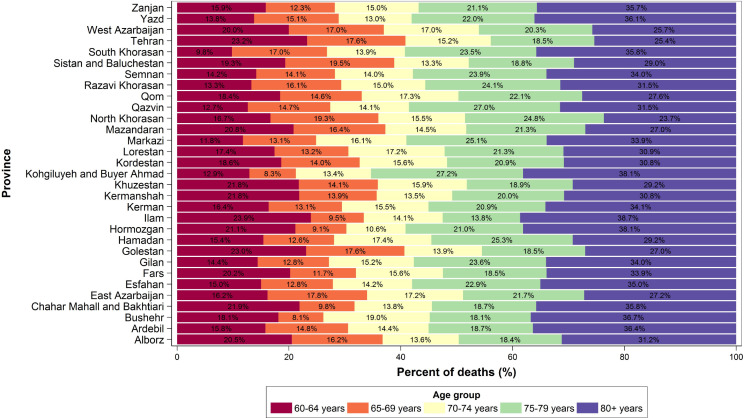
Percentage of burn mortality based on age groups among provinces in 2015

In 2015, the highest percentage of deaths occurred amongst those aged 80 years or more in all provinces except North Khorasan, the percentage ranged 23.7%-38.7%. In almost half of the provinces, the second rank belonged to the 75-79 years age group whose percentage of death ranged 13.8-27.2%. In other age groups, the percentage of burn mortality was different among different provinces, 70-74 years age group ranged 10.6-19.0%, 65-69 years age group ranged 8.1-19.3%, and 60-64 years age group ranged 9.8-21.9% (Figure 1).

The age-standardized mortality rate was more than 8.54 /100,000 people among men in seven provinces (South Khorasan, North Khorasan, Hormozgan, Ilam, Kohgiluyeh and Buyer Ahmad, Golestan, and Semnan), which was the same for women except for Bushehr replacing Semnan. 

At the national level, from 1990 to 2011, the age-standardized burn mortality per 100,000 was higher among females than males. However, in the final four years of the study period (i.e. from 2012 to 2015), males’ age-standardized burn mortality rate was a little higher than females (Supplementary Figure 1).

At the national level, the mortality rate for females in 1990 and 2015 were 21.5 and 4.5, respectively, and for males the rates were 14.0 and 4.6 per 100,000, respectively (Table 1). 

At the provincial level, in 1990, the highest and lowest mortality rates per 100,000 were 42.8 among females of North Khorasan and 5.7 among males of Alborz province, respectively. Supplementary Table1 indicates national and subnational mortality rates due to burns per 100,000 population in 2015. The lowest and highest mortality rates belonged to Tehran and South Khorasan with mortality rates of 0.9 and 12.6 among males and 1.2 and 11.9 per 100,000 among females, respectively.

At the national level, the APC of burn mortality rate per 100,000 people, from 1990 to 2015, was -6.1% in females and -4.4% in males (Table 1). In 31 provinces, the highest APC was found amongst Razavi Khorasan females (-8.6%) and the lowest APC belonged to Hormozgan males (-1.7%) (Supplementary Table 1).

**Figure 2:**
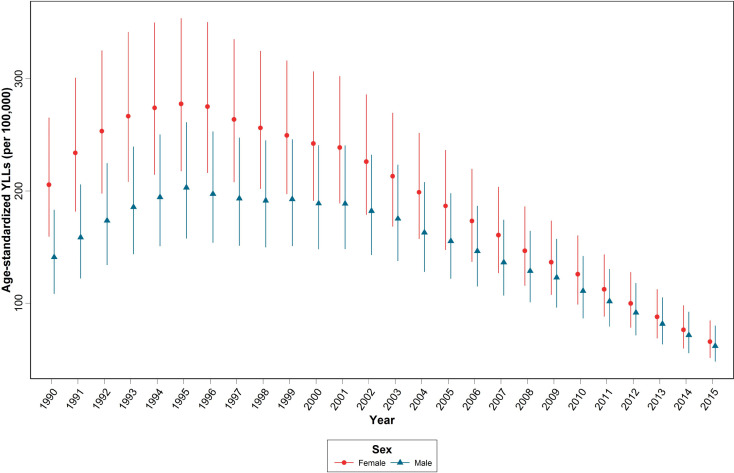
Time trend of age-standardized years of life lost (YLLs) due to burn mortality per 100,000 females and males at the national level during 1990-2015

At the national level, as a result of the burn mortality among those aged 60+, a total of 4586 YLLs (2070 years in males and 2516 yrs. in females) in 2015 and 4862 YLLs (2659 in females and 2203 in males) in 1990 were estimated. Figure 2 indicates the time trend of age-standardized YLLs burn mortality per 100,000 females and males at the national level from 1990 to 2015.

At the subnational level, the province with the largest estimated burden in terms of YLLs was Razavi Khorasan, with 186 YLLs for females and 173 for males in 2015. The lowest YLLs were 17 for males in Alborz and 23 for females in Qom province (Table 2). Overall, in our study, burn mortality indexes in females were higher than males in 4 measurements of 31 provinces 1) based on the age-standardized rate in 21 provinces 2), based on the 60+ year-olds’ mortality rate in 19 provinces 3), based on the 60+ year-olds’ number in 23 provinces and based on the rate of years of life lost /100,000 in 21 provinces (Table 2). 

The age-standardized rate of YLLs due to burn mortality (per 100,000) at the subnational level in 2015 indicated that in only 10/31 provinces this rate in males was higher than females. 

At the national level, the APC of YLLs between 1990 and 2015 was 4.3% in females and 3% in males (Table 1). Among 31 provinces, the highest APC of YLLs (-6.6%) was in females of Esfahan and the least (-0.5%) belonged to Hormozgan males (Table 2).

## 4. Discussion

The findings show a decrease in both burn-related mortality rate (17.4 per 100,000 to 4.54 per 100,000) and YLLs (4962 to 4586 YLLs) during the 26-year study period from 1990 to 2015. The male/female ratio was 0.8 and 0.88 in 1990 and 2015, respectively.

Existing policies are in place for burn mortality reduction at the national level are improvement of health and treatment, increased education in television, news, and poster media development of specialized burn hospitals and specialist burn wards in the hospitals, standard registration of trauma data including burn, development of research burn centers and safety improvement of house and workplaces. Safer equipment such as stoves and electrical heater etc. 

This trend is in line with several studies. WHO reported that 12,057 fire-related deaths occurred among 75+ year-old people in 31 European countries, from 2005 to 2014. The mean fire-related mortality rate was 2.86 per 100,000 population, but the range significantly varied between 0.55 in Iceland to 14.65 in Latvia (12). In the United States, the incidence of burn injury has reduced from 215 to 140 per 100,000 from 1990 to 2016. However, during this time, burn-related death has been static. Alaska has had the highest rate of burn incidents in 1990 and 2016. Similar to studies in other countries, the reductions have not been equally distributed throughout the US (32). In Spain, a study showed that 60.1% of death victims in fires are people > 65 years old (the percentage of the population> 65 aged was 18.3%). This means that the RR of dying in a fire is increased by 3.3 for the old population (33). 

In a Dutch study carried out by Jan Dokter et al., most of patients who died during the study were over 65 years old. Of 2730 patients in 2 burn centers, 88 patients died as a result of their burn injury. The overall mortality rate was 3.2%. Mortality in burn patients decreased during the study period (34).In the Netherlands, adult patients admitted to one of the three Dutch burn centers from 2009 to 2015 were included in a study. Burn patients ≥ 65 years of age were studied as the elderly, and adults in the age group 18–64 years were the reference group. The results indicated that death quickly increased with higher age and its rate was the highest in the 85+ year-old age group (23.8%). Substantial differences were observed in hospital release between the old people and the reference category (21).

Inconsistent with our study, among burn patients who were admitted to a major burn center in southwest China from 2011 to 2015, the mortality did not clearly decrease during this time (35). 

The age-standardized mortality rate of the burn was more than 8.58 per 100,000 females and males in 6 and 7 provinces of Iran in 2015, respectively, which is very high.

Compared to our study, according to WHO estimates, among 310,000 burn-related mortalities around the world, the majority occurred in LMICs with a rate of 4.8/100,000 people per year. While 29,000 deaths happened in the Eastern Mediterranean Regional Office (EMRO) of WHO (which is one of WHO's six regional offices in the world and includes 22 countries and territories in the Middle East) with a mortality rate of 5.6 per 100,000. A study in Kuwait, one of the three HICs of EMRO, indicated that all-age mortality rate was 0.6 per 100,000 /year (36). The burn mortality rate reported in 16 studies in Iran was from 1.4 - 9.7 per 100,000 in all age groups(14).

The male/female ratio in 1990 and 2015 was 0.8 and 0.88, respectively. Although from 1990 to 2015, age-standardized burn mortality per 100,000 at the national level in females was more than in males in most years, in the last years of the study, this index became a little higher in males. In 2015, the mortality rate per 100,000 is almost the same in females and males (4.5 VS 4.6). Various studies on burn injuries in Iran and other middle-income countries have shown that the highest rate of burns occur at home and women are more at risk than men at home. It seems that preventive measures and promotion of public health in these years have reduced mortality by affecting the at-risk group.

In our study, the percentage distribution of burn mortality in 5 age groups including 60-64, 65-69, 70-74, 75-79, and 80+ years in females ranged from 2.4-10.9 and in males the range was 2.5-10.2 per 100,000. The majority of burn mortalities at the national and the provisional levels were amongst the 80+ year-old age group. 

Although in most countries, the burn mortality rate is different between the two sexes. In the United States, in a study among 6512 patients aged > 70 years, admitted to a Burn Center between 1995 and 2011 (17 years), outcomes of burn injuries indicated that the mean age of the patients was 80.4 ± 6.7 years, and female patients (58%) were more than males (37).

Regarding the sex differences, a study on 1044 hospitalized burn patients in Iran indicated that, although the number of hospitalized burn patients was higher in males, mortality of burn was higher in females (22%) than in males (12.5%) (18). Another study indicated that the rate of women’s death as a result of the burn was 2.2 times higher than men’s (38). In Bangalore, India, a ten-year retrospective study of mortality among elderly burn victims in a medical college hospital indicated that the majority of the victims were women (39). Literacy and beliefs about cooking at home by women can explain this discrepancy.

Similarly, a study in a referral burn center in Iran indicated that burn mortality in females in the final age group was significantly higher than in males(15). The researcher believes that because the life expectancy in females (76.87 years) is 2.2 years higher than in males (74.67 years) in Iran, excessive burn mortality in females older than 65-years may be related to the greater number of females in this age category(15). Another study reported significantly rising mortality of 1.03 times with a one-year increase in age and 1.02 increase with a one-day rise in hospital stay (38). These are consistent with our study, which indicated that when the age increases from 60 to more than 80, the mortality rate for burn increases.

Mandell et al., in a study of burn mortality with 2 years follow-up, compared patients aged 45 - 54 years with the older age category. Results indicated an increased mortality risk with an odds ratio (OR) of 1.53 for the 55 - 64 years age group, 2.51 for the 65-74 years age group, and 2.90 for the ≥ 75 years age group.(40).

The present study shows that the lowest and highest mortality rates were 0.9 vs. 12.6 per 100,000 in males and 1.2 vs. 11.9 in females. The outcome of burns vastly differs in different countries and also in different provinces of Iran. Researchers found that these differences may be attributed to the financial, cultural, geographical, and social situations, and lifestyle of the study populations (14, 19). Our results indicated that during the study period some provinces such as Tehran had a lower mortality rate compared to others such as Sistan Va Baluchistan. This may be related to better treatment facilities and socio-economic situation of people in Tehran.

APC of burn mortality rate during 1990 – 2015 has been -6.1 per 100,000 in females and -4.4 per 100,000 in males. This decline indicates better preventive measurement, which was higher in females than in males. At the national level, as a result of burn mortality in ages ≥60 years a total of 4586 years of life was lost in 2015. The YLLs due to burning mortality increased from 60 to 80+ year-olds. Among age groups, the majority of YLLs belong to those aged 80 years and higher, similar to the mortality rate. But the direction is not linear and has some curvatures.

APC of YLLs between 1990 and 2015 was -4.3% in females and -0.3 % in males. This indicates positive changes in survival and better preventive measurement in women compared to men. 

The highest APC for YLLs /100,000 was -6.6%, which belonged to females of Esfahan province and the lowest was -0.5%, which belonged to males in Hormozgan, as a border province. The key points here are the variation of burn mortalities by regions in specific age groups. This is also indicated in other countries such as Europe and USA(12, 32).

To further reduce the burn mortality rate in Iran, reinforcing existing burn prevention and interventional measures are suggested, especially in border provinces with higher mortality rates. Also, paying attention to the distribution of burn injuries with more detail in older people by cause, types, burn sites, comorbidity, total body surface area, length of hospitalization, and severities are recommended for future studies.

***4.1. Clinical implications for health managers and policymakers***

The findings could be used to plan and implement new programs in Iran in order to make better use of limited resources, such as improving the registration of burn injuries and health care, providing advanced treatment, providing more ambulances, and providing more education in cities with higher mortality rates. Paying special attention to preventive measures in HICs with lower burn-related mortality and morbidity, such as Best Practice for Skin and Wound Management, is recommended.

**Table 1:** National mortality and years of life lost (YLLs) due to burn in 1990 and 2015 with annual percentage change (APC) between 1990 and 2015

**Measure**	**Sex**	**Year**	**2015**							**1990 to 2015**	
			**Rate** **(per 100,000)**					**Rate** **(per 100,000)**	**Number**	**APC (%)**	
										**Rate**	**Number**
			**60-64**	**65-69**	**70-74**	**75-79**	**80+**	**Elderly (60+)**			
Death	Female	1990	12.1 (9.3-15.6)	15.9 (12.3-20.5)	24.1 (18.7-31.1)	44.1 (34.1-56.9)	51.8 (40.1-66.9)	21.5 (16.6-27.7)	290 (225-374)	-6.1	-2.1
		2015	2.4 (1.9-3.1)	2.9 (2.3-3.7)	4.2 (3.3-5.4)	6.0 (4.6-7.8)	10.9 (8.5-14.1)	4.5 (3.5-5.8)	173 (134-222)		
	Male	1990	7.8 (6.0-10.2)	12.6 (9.7-16.3)	15.4 (11.9-20.0)	28.1 (21.5-36.7)	36.5 (28.2-47.1)	14.0 (10.8-18.2)	233 (179-303)	-4.4	-1.7
		2015	2.5 (1.9-3.2)	3.3 (2.5-4.2)	4.1 (3.2-5.3)	5.6 (4.3-7.3)	10.2 (7.9-13.3)	4.6 (3.5-5.9)	153 (118-198)		
YLLs	Female	1990	181.5 (140.5-234.3)	182.2 (141.1-234.7)	205.5 (159.5-265.2)	262.9 (203.5-339.5)	237.5 (183.9-306.6)	196.9 (152.5-254)	2,659 (2,060-3,430)	-4.3	-0.2
		2015	57.2 (44.6-73.6)	56.0 (43.7-72.0)	64.4 (50.4-82.4)	71.0 (55.0-91.9)	98.2 (76.4-126.5)	65.7 (51.1-84.5)	2,516 (1,959-3,239)		
	Male	1990	114.3 (87.7-148.8)	140.0 (107.6-181.6)	130.3 (100.3-169.4)	169.2 (129.3-221.1)	177.7 (137.4-229.4)	132.8 (102.1-172.6)	2,203 (1,693-2,863)	-3.0	-0.2
		2015	53.5 (41.4-68.9)	57.3 (44.4-74.2)	58.4 (45.3-75.2)	62.8 (48.4-81.8)	88.8 (68.4-115.0)	61.7 (47.7-79.8)	2,070 (1,600-2,676)		

* Data in parentheses are 95% uncertainty intervals.

**Table 2:** Subnational years of life lost (YLLs) due to burn in 2015 with annual percentage change (APC) between 1990 and 2015

**Location**	**Sex**	**2015**							**1990 to 2015**	
		**Rate** **(per 100,000)**					**Rate** **(per 100,000)**	**Number**	**APC (%)**	
									**Rate**	**Number**
		**60-64**	**65-69**	**70-74**	**75-79**	**80+**	**Elderly (60+)**			
Alborz	Female	24.6 (18.4-33.2)	24.7 (18.7-32.8)	22.1 (16.6-29.1)	27.6 (21.0-36.7)	44.0 (33.6-57.3)	26.6 (20.1-35.4)	30 (23-40)	-4.1	2.4
	Male	14.9 (11.1-19.7)	15.2 (11.2-20.0)	14.3 (10.7-18.9)	15.9 (11.9-21.2)	22.2 (16.7-29.6)	15.7 (11.7-20.8)	17 (13-22)	-5.1	0.3
Ardebil	Female	92.9 (74.9-114.6)	98.4 (78.2-122.5)	118.5 (95.1-147.3)	93.8 (75.2-116.0)	157.2 (125.3-197.3)	107.2 (85.8-133.2)	64 (52-80)	-2.4	1.0
	Male	88.5 (70.7-110.8)	79.2 (63.1-99.0)	79.0 (62.3-99.2)	72.5 (57.7-90.9)	89.0 (70.2-111.6)	82.4 (65.4-103.2)	45 (36-57)	-3.1	-1.2
Bushehr	Female	78.9 (62.1-99.7)	88.1 (68.6-113.7)	107.0 (84.4-135.0)	152.1 (119.1-193.1)	165.7 (130.3-209.9)	104.1 (81.8-132.0)	46 (36-58)	-4.5	-0.2
	Male	73.5 (57.7-94.1)	73.3 (55.4-96.5)	99.7 (76.9-127.6)	111.1 (86.2-142.4)	120.1 (93.4-153.7)	91.6 (71.1-117.7)	33 (26-43)	-2.4	0.5
Chahar Mahal and Bakhtiari	Female	118.9 (95.1-148.6)	101.9 (80.7-127.8)	117.1 (92.7-147.4)	129.2 (102.5-161.4)	158.7 (126.7-198.9)	123.3 (98.2-154.5)	54 (43-67)	-2.7	1.3
	Male	100.3 (79.6-126.6)	88.7 (68.2-114.3)	92.4 (72.1-117.8)	98.8 (77.9-124.5)	141 (111.1-180.4)	103.9 (81.8-132.1)	39 (31-49)	-3.0	-0.7
East Azarbaijan	Female	53.9 (42.9-69.0)	58.1 (45.9-73.4)	61.1 (48.6-77.9)	58.3 (45.4-76.3)	77.0 (60.4-98.2)	59.9 (47.3-76.6)	127 (100-163)	-5.0	-1.4
	Male	51.5 (40.2-65.1)	62.1 (48.8-79.0)	52.1 (40.9-66.0)	63.2 (48.8-81.4)	80.7 (62.5-103.4)	60 (46.8-76.5)	112 (88-143)	-3.0	-0.9
Esfahan	Female	32.1 (24.0-42.6)	32.7 (24.7-43.4)	38.1 (28.9-50.6)	46.7 (34.9-61.9)	49.7 (37.8-65.6)	38 (28.7-50.4)	105 (79-140)	-6.6	-2.7
	Male	43.3 (32.4-57.3)	41.1 (30.3-55.5)	46.2 (34.8-61.3)	45.3 (33.3-61.7)	69.6 (51.4-93.9)	47.0 (34.9-62.9)	118 (88-159)	-4.3	-1.2
Fars	Female	60.9 (47.5-77.8)	58.4 (45.9-74.4)	65.6 (51.6-83.0)	69.1 (54.1-89.8)	104.8 (81.3-135.8)	68.6 (53.6-87.9)	163 (127-209)	-3.9	0.0
	Male	56.9 (43.9-73.1)	65.2 (50.5-84.1)	67.7 (52.9-86.1)	75.2 (58.5-97.8)	77.7 (59.3-102.0)	66.3 (51.2-85.6)	137 (106-177)	-2.2	0.7
Gilan	Female	49.1 (38.9-62.3)	59.4 (47.2-74.3)	73.8 (58.2-93.6)	87.0 (66.6-113.2)	132.7 (103.5-170.9)	72.3 (56.7-92.4)	125 (98-160)	-3.9	-0.5
	Male	48.0 (37.8-60.1)	51.2 (41.4-64.4)	55.5 (44.2-69.2)	59.0 (46.3-74.9)	78.2 (61.0-99.0)	56.0 (44.3-70.5)	86 (68-108)	-3.1	-0.3
Golestan	Female	96.4 (76.1-121.3)	86.3 (68.7-108.8)	84.1 (67.1-105.9)	84.8 (67.2-106.8)	142.8 (113-179.1)	95.1 (75.4-119.6)	74 (58-93)	-3.6	0.5
	Male	86.3 (68.0-108.7)	89.0 (70.3-111.8)	91.9 (72.0-117.0)	78.2 (62.0-98.7)	120.9 (94.4-152.0)	90.2 (71.0-113.7)	58 (46-74)	-2.9	-0.4
Hamadan	Female	94.3 (76.5-116.5)	73.6 (59.9-91.3)	98.8 (80.5-122.4)	138.1 (111.0-173.9)	161.4 (129.6-200.1)	105.8 (85.6-131.4)	109 (88-136)	-2.5	1.0
	Male	77.1 (62.0-95.2)	83.1 (66.9-103.2)	93.4 (75.6-117.0)	89.1 (70.8-111.8)	115.7 (92.1-144.4)	89.5 (71.7-111.5)	75 (60-94)	-2.4	-0.4
Hormozgan	Female	121.9 (93.6-157.9)	112.9 (86.7-146.9)	156.9 (120.7-206.5)	183.3 (138.5-241.1)	194.1 (147.7-255.4)	146.1 (111.6-190.9)	86 (66-113)	-3.2	0.9
	Male	109.8 (84.7-142.6)	128.7 (98.7-170.1)	141.5 (106.1-187.9)	144.3 (107.9-191.5)	196.5 (147.7-261.2)	140.9 (106.8-185.8)	72 (55-96)	-0.5	2.1
Ilam	Female	125.7 (100.4-158.3)	116.1 (90.1-149.3)	155.6 (120.0-201.4)	126.7 (99.1-161.6)	204.5 (159.7-259.7)	138.3 (108.7-175.9)	37 (29-46)	-4.3	0.2
	Male	117.5 (93.1-149.1)	109.7 (83.5-144.6)	123.9 (97.2-160.0)	122.5 (96.6-159.3)	134.3 (106.0-170.4)	121.8 (95.8-156.2)	29 (23-37)	-3.1	-0.6
Kerman	Female	65.2 (52.1-80.9)	67.9 (54.7-83.7)	91.3 (72.9-114.7)	91.4 (72.1-116.2)	125.0 (99.1-158.7)	83.1 (66.2-104.1)	108 (86-135)	-3.7	0.5
	Male	60.8 (48.7-75.4)	72.1 (57.2-89.7)	75.2 (60.5-93.8)	75.2 (58.3-96.0)	102.6 (80.7-131.1)	74.8 (59.2-94.0)	83 (66-105)	-2.3	0.3
Kermanshah	Female	90.4 (72.3-112.6)	96.0 (76.9-119.7)	80.7 (64.4-102.4)	94.4 (75.9-117.3)	131.6 (105.9-162.7)	95.2 (76.2-118.7)	101 (81-126)	-3.7	0.8
	Male	67.5 (54.1-84.5)	72.0 (57.6-90.8)	76.1 (60.7-95.4)	81.5 (64.2-102.6)	106.7 (85.0-133.3)	78.3 (62.5-98.3)	69 (55-87)	-2.4	-0.1
Khuzestan	Female	51.1 (39.7-65.6)	48.7 (37.7-61.8)	52.4 (41.2-66.6)	57.9 (45.7-73.9)	75.4 (59.9-95.2)	54.6 (42.7-69.6)	96 (75-122)	-4.9	-1.1
	Male	43.4 (33.7-55.9)	45.4 (35.7-57.9)	46.3 (36.4-59.0)	43.0 (33.7-54.8)	49.1 (38.2-62.8)	45.0 (35.2-57.6)	68 (53-87)	-3.0	-0.2
Kohgiluyeh and Buyer Ahmad	Female	104.1 (80.9-134.0)	116.4 (88.1-153.0)	101.1 (78.9-131.0)	208.5 (161.8-273.1)	233.0 (178.9-303.3)	142.0 (109.7-184.7)	36 (28-47)	-3.4	0.7
	Male	91.9 (70.0-119.8)	112.3 (84.3-152.1)	129.3 (97.4-172.5)	152.7 (117.0-201.5)	148.0 (113.2-193.9)	126.5 (96.2-167.0)	31 (24-41)	-3.3	0.1
Kordestan	Female	70.7 (56.5-87.2)	73.2 (59.3-91.1)	90.1 (73.2-111.5)	90.4 (72.8-112.4)	122.9 (99.7-152.2)	83.8 (67.5-103.7)	62 (50-76)	-4.2	-0.3
	Male	54.6 (43.9-68.0)	62.4 (49.9-77.6)	63.1 (50.4-78.8)	60.8 (48.8-76.3)	79.6 (63.5-99.3)	62.3 (49.9-77.7)	41 (33-51)	-4.1	-2.2
Lorestan	Female	73.7 (59.6-91.7)	91.9 (74.2-113.6)	87.0 (70.4-107.6)	101.8 (81.8-125.7)	115.3 (92.6-143.2)	89.1 (71.8-110.4)	75 (61-93)	-4.0	-0.1
	Male	66.3 (52.7-83.4)	76.5 (60.9-96.1)	68.8 (55.2-86.6)	64.9 (51.7-81.7)	94.2 (75.1-118.3)	73.0 (58.2-91.8)	52 (41-65)	-3.1	-1.1
Markazi	Female	61.7 (48.8-78.1)	71.8 (57.5-90.3)	74.0 (58.9-91.7)	88.7 (70.1-111.7)	140.6 (112.1-176.7)	82.1 (65.3-103.2)	71 (57-90)	-4.8	-1.2
	Male	62.7 (49.7-79.5)	83.7 (66.3-105.2)	75.3 (59.9-94.8)	90.4 (70.7-114.8)	111.6 (87.9-140.2)	83.0 (65.5-104.8)	60 (47-76)	-3.1	-1.0
Mazandaran	Female	60.1 (45.9-77.9)	64.2 (49.5-83.9)	68.3 (52.7-88.4)	66.5 (49.9-88.0)	119.8 (91.2-158.3)	69.7 (53.3-91.2)	131 (100-171)	-3.8	0.3
	Male	55.9 (43.1-72.4)	66.2 (51.1-85.3)	56.9 (44.0-72.6)	56.5 (43.5-75.0)	79.1 (59.9-104.8)	61.1 (47.0-79.4)	103 (79-134)	-3.1	0.1
North Khorasan	Female	126.6 (99.7-164.2)	131.2 (100.8-169.7)	159.2 (122.8-204.7)	190.9 (149.2-246.1)	167.5 (131.9-212.9)	147.5 (114.9-190.1)	61 (47-78)	-3.4	0.6
	Male	148.1 (112.2-192.2)	145.7 (112.4-188.2)	134.2 (102.4-175.8)	153.0 (118.7-195.6)	181.9 (138.9-236.1)	151.1 (115.8-195.5)	51 (39-67)	-2.4	-0.3
Qazvin	Female	59.3 (47.4-74.7)	70.6 (56.9-88.1)	86.1 (69.3-107.2)	84.7 (67.7-105.4)	146.5 (118-181.8)	81.6 (65.5-101.8)	49 (40-62)	-3.7	0.2
	Male	62.4 (49.1-78.7)	70.9 (56.5-89.9)	82.5 (65.0-103.7)	97.4 (77.4-121.6)	137.7 (109.6-171.1)	85.2 (67.5-106.9)	45 (35-56)	-2.6	0.0
Qom	Female	42.8 (31.5-57.7)	42.2 (31.2-56.9)	51.5 (38.4-69.1)	53.7 (40.2-71.4)	73.3 (54.9-97.0)	49.4 (36.7-66.2)	23 (17-31)	-4.1	-0.2
	Male	64.7 (48.0-88.2)	70.5 (51.2-96.2)	61.5 (44.5-84.3)	63.5 (46.5-86.2)	91.6 (67.9-125.0)	68.1 (50.0-92.9)	29 (21-39)	-2.4	0.6
Razavi Khorasan	Female	50.7 (39.2-65.1)	55.5 (43.1-71.1)	61.1 (47.4-78.9)	70.7 (53.6-92.9)	107.1 (82.8-137.9)	64.6 (49.8-83.4)	186 (143-240)	-6.0	-2.3
	Male	61.6 (48.0-79.2)	60.5 (47.5-77.9)	69.5 (54.0-88.6)	66.0 (49.6-88.0)	122.1 (92.8-160.3)	71.4 (54.9-92.8)	172 (132-223)	-3.0	-1.0
Semnan	Female	73.6 (57.0-95.9)	84.8 (66.3-109.3)	91.0 (71.3-116.6)	88.7 (70.3-112.3)	142.3 (112.4-181.0)	91.3 (71.5-117.1)	32 (25-41)	-4.6	-1.4
	Male	89.0 (67.2-118.1)	92.2 (70.4-119.8)	104.0 (79.6-136.2)	129.3 (101.2-165.1)	155.6 (121.5-199.9)	109.5 (84.3-142.3)	34 (26-44)	-2.7	-0.5
Sistan and Baluchestan	Female	111.9 (86.0-146.4)	142.3 (109.5-187.1)	137.6 (106.7-178.5)	109.8 (84.6-143.0)	177 (134.4-235.9)	131 (100.6-171.8)	97 (74-127)	-2.6	1.4
	Male	122.0 (92.7-159.0)	142.1 (107.3-185.0)	147.6 (113.2-193.5)	125.8 (95.9-165.8)	144.5 (109.2-190.0)	134.6 (102.2-176.1)	89 (68-117)	-1.2	0.8
South Khorasan	Female	126.4 (96.6-165.0)	169.9 (132.9-220.2)	198.5 (150.1-259.4)	175.3 (135.3-227.8)	226.8 (173.2-298)	175.7 (135-229.1)	62 (47-80)	-2.9	-0.4
	Male	143.2 (108.6-186.1)	163.7 (125.3-216.1)	142.5 (107.3-187.5)	140.5 (109.3-185.3)	230.5 (176.7-300.4)	163.3 (125.0-214.2)	52 (40-68)	-1.9	-0.9
Tehran	Female	20.7 (14.3-30.2)	16.4 (11.2-24.2)	17.6 (12.1-25.0)	21.1 (14.5-31.2)	29.7 (20.4-43.7)	20.3 (14.0-29.7)	140 (96-205)	-5.0	-0.2
	Male	15.6 (10.7-22.5)	15.9 (10.7-23.3)	15.0 (10.4-22.0)	15.1 (10.1-22.7)	17.7 (11.9-26.1)	15.7 (10.7-23.0)	98 (67-144)	-4.8	-0.9
West Azarbaijan	Female	52.1 (42.0-65.5)	52.0 (41.5-65.7)	67.9 (54.1-84.3)	56.0 (44.0-71.1)	88.3 (70.4-110.5)	59.7 (47.7-75.1)	90 (72-114)	-3.7	0.6
	Male	53.2 (42.8-66.8)	60.2 (48.1-75.0)	55.6 (44.9-69.0)	51.0 (40.5-63.8)	61.1 (48.5-76.3)	55.7 (44.6-69.6)	69 (55-86)	-2.2	0.2
Yazd	Female	61.8 (48.9-79.1)	68.9 (54.2-87.4)	73.1 (57.6-91.8)	72.7 (57.7-91.3)	80.8 (64.1-102.2)	71.2 (56.3-90.2)	43 (34-55)	-4.9	-1.1
	Male	82.1 (63.1-106.2)	94.3 (72.9-123.5)	82.9 (64.6-106.3)	90.9 (70.6-116.8)	123.4 (96.6-156.1)	93.0 (72.1-119.7)	44 (34-57)	-3.6	-1.2
Zanjan	Female	83.5 (63.9-109.9)	77.8 (58.9-104.2)	90.6 (69.0-119.5)	111.8 (84.4-148.4)	208.7 (157.9-276.3)	102.6 (77.9-135.9)	55 (42-73)	-1.8	1.8
	Male	81.1 (59.7-109.0)	63.1 (47.5-83.8)	79.4 (58.1-107.1)	63.5 (47.9-85.2)	119.4 (89.5-160.1)	78.5 (58.4-105.3)	35 (26-47)	-1.8	0.2

* Data in parentheses are 95% uncertainty intervals.

## 5. Limitation

One limitation of this study was the lack of some variables such as duration of hospitalization, inhalation injury, and total body surface area. Using the standardized registration system of WHO for trauma, including burns, we can overcome this problem in future studies.

 Another limitation is that burns will vary between regions within a province and we are masking that by providing an average, the same as a national average. Interventional studies with regard to preventive measures such as household safety interventions, improvement in health care, advanced treatment, increase in access to …., providing more ambulances, and more education should be implemented in cities with high mortality rate. 

The strengths of this study were the validity of data resources and extended data across the country. To the best of our knowledge, this is the first study focused on the epidemiological characteristics of burn mortality in people ≥ 60 years in Iran, which provides information and better understanding for planning preventive measures.

## 6. Conclusion

In our study, burn mortality rate and its related YLLs in people ≥ 60 years decreased during the studied 26 years, but is still high in comparison with HICs. Among people≥ 60 years, premature mortality due to burning injuries were more prevalent among those with older age and those residing in border provinces. Also, difference between provinces was high in two epidemiological indexes. 

## 7. Declarations

### 7.1. Acknowledgments

The authors would like to express their appreciation for the contributions of the deputy for the health of Iran’s Ministry of Health and Medical Education. We would like to appreciate Dr. Farshad Farzadfar, Chair of the Non-Communicable Diseases Research Center (NCDRC) of Endocrinology and Metabolism Research Institute of Tehran University of Medical Sciences, who is the principal investigator of the NASBOD study. The authors would like to thank Dr. Mohammadreza Zafarghandi, Chair of Sina Trauma and Surgery Research Center, Tehran University of Medical Science. Also, the authors would like to thank Dr. Mehrdad Azmin and the staff at NCDRC for their wholehearted cooperation. 

### 7.2. Author contribution

All the authors of this study met the standard criteria of authorship based on the recommendations of the International Committee of Medical Journal Editors.

### 7.3. Funding

This work was funded by the Iranian Ministry of Health and Medical Education [grant number: 1391-01-101-150] and Sina Trauma and Surgery Research Center [number: 98-01-38- 41639]. 

### 7.4. Conflict of Interest

None declared.

## References

[1] Forbinake NA, Ohandza CS, Fai KN, Agbor VN, Asonglefac BK, Aroke D (2020). Mortality analysis of burns in a developing country: a CAMEROONIAN experience. BMC Public Health.

[2] Jonsson A, Runefors M, Särdqvist S, Nilson F (2016). Fire-related mortality in Sweden: temporal trends 1952 to 2013. Fire technol.

[3] Duci SB, Arifi HM, Ahmeti HR, Zatriqi VK, Buja ZA, Hoxha ET (2015). Outcomes of older adults with burn injury: University Clinical Center of Kosovo. World J  Plast  Surg.

[4] Simsek ME, Özgenel GY, Kahveci R, Akın S, Özbek S, Tufan F (2015). Outcomes of elderly burn patients requiring hospitalization. The Aging Male.

[5] Hashmi A, Ibrahim-Zada I, Rhee P, Aziz H, Fain MJ, Friese RS (2014). Predictors of mortality in geriatric trauma patients: a systematic review and meta-analysis. J Trauma Acute Care.

[6] Ahrens M (2017). Trends and patterns of US fire loss.

[7] Center for disease Control and prevention ,CDC, Prevention (2016). Trends in current cigarette smoking among high school students and adults, United States, 1965–2014.

[8] Clare J, Kelly H (2017). Fire and at risk populations in Canada. Analysis of the Canadian National Fire Information Database Abbotsford, BC.

[9] Duke JM, Boyd JH, Rea S, Randall SM, Wood FM (2015). Long-term mortality among older adults with burn injury: a population-based study in Australia. Bull  World Health Organ.

[10] Firchal EW, Sjoberg F, Fredrikson M, Pompermaier L, Elmasry M, Steinvall I (2021). Long-term survival among elderly after burns compared with national mean remaining life expectancy. Burns.

[11] Sharma NP, Duke JM, Lama BB, Thapa B, Dahal P, Bariya ND (2015). Descriptive epidemiology of unintentional burn injuries admitted to a tertiary-level government hospital in Nepal: gender-specific patterns. APJPH.

[12] Nilson F, Lundgren L, Bonander C (2020). Living arrangements and fire-related mortality amongst older people in Europe. Int J Inj Contr Saf Promot.

[13] Peck M, Pressman MA (2013). The correlation between burn mortality rates from fire and flame and economic status of countries. Burns.

[14] Sadeghi-Bazargani H, Mohammadi R (2012). Epidemiology of burns in Iran during the last decade (2000–2010): review of literature and methodological considerations. Burns.

[15] Khadem-Rezaiyan M, Aghajani H, Ahmadabadi A, Zanganeh M, Tavousi SH, Sedaghat A (2020). Epidemiology of severe burns in North-East of Iran: How is the burn size different in a developing country from developed ones?. Burns Open.

[16] Abdolkarimi L, Taftachi F, Hayati F, Mehrpisheh S, Moghadam N ( 2018). Epidemiologic study of burns in elderly people over 60 years old. Tehran Univ  Med  J.

[17] Sarbazi E, Yousefi M, Khami B, Ettekal-Nafs R, Babazadeh T, Gaffari-Fam S (2019). Epidemiology and the survival rate of burn-related injuries in Iran: a registry-based study. Burns Fire Disasters.

[18] Ahmadabadi A, Aghajani H, Khadem Rezaiyan M, Zanganeh M, Tavousi S, Hadianfar A (2019). Spatial analysis of hospitalized burn injuries and related risk factors in Mashhad, Iran. Iran  J  Epidemiology.

[19] World Health Organisation https://www.who.int/news-room/fact-sheets/detail/burns.2020.

[20] World Health Organisation http://www.who.int/mediacentre/factsheets/fs404/en/2015;September2015.

[21] Goei H, van Baar ME, Dokter J, Vloemans J, Beerthuizen GI, Middelkoop E (2020). Burns in the elderly: a nationwide study on management and clinical outcomes. Burns Trauma.

[22] Wearn C, Hardwicke J, Kitsios A, Siddons V, Nightingale P, Moiemen N (2015). Outcomes of burns in the elderly: revised estimates from the Birmingham Burn Centre. Burns.

[23] Jeschke MG, Pinto R, Costford SR, Amini-Nik S (2016). Threshold age and burn size associated with poor outcomes in the elderly after burn injury. Burns.

[24] World Health Organisation https://www.WHO.int/data/gho/indicator-metadata-registry/imr-details/4645.2022.

[25] Farzadfar F, Delavari A, Malekzadeh R, Mesdaghinia A, Jamshidi HR, Sayyari Aa et al (2014). NASBOD 2013: design, definitions, and metrics (study protocol). Arch Iran Med.

[26] Parsaeian M, Farzadfar F, Zeraati H, Mahmoudi M, Rahimighazikalayeh G, Navidi I (2014). Application of spatio-temporal model to estimate burden of diseases, injuries and risk factors in Iran 1990–2013. Arch Iran Med.

[27] Sheidaei A, Gohari K, Kasaeian A, Rezaei N, Mansouri A, Khosravi A (2017). National and subnational patterns of cause of death in iran 1990-2015: Applied Methods. Arch Iran Med.

[28] Mohammadi Y, Parsaeian M, Farzadfar F, Kasaeian A, Mehdipour P, Sheidaei A (2014). Levels and trends of child and adult mortality rates in the Islamic Republic of Iran, 1990-2013; protocol of the NASBOD study. Arch Iran Med.

[29] Mohammadi Y, Parsaeian M, Mehdipour P, Khosravi A, Larijani B, Sheidaei A (2017). Measuring Iran's success in achieving Millennium Development Goal 4: a systematic analysis of under-5 mortality at national and subnational levels from 1990 to 2015. Lancet Glob Health.

[30] Mehdipour P, Navidi I, Parsaeian M, Mohammadi Y, Moradi LM, Rezaei DE (2014 ). Application of Gaussian Process Regression (GPR) in estimating under-five mortality levels and trends in Iran 1990-2013, study protocol. Arch Iran Med.

[31] Sadeghian F, Moghaddam SS, Saadat S, Niloofar P, Rezaei N, Amirzade-Iranaq MH (2019). The trend of burn mortality in Iran—a study of fire, heat and hot substance-related fatal injuries from 1990 to 2015. Burns.

[32] Crowe CS, Massenburg BB, Morrison SD, Naghavi M, Pham TN, Gibran NS (2019). Trends of burn injury in the United States: 1990 to 2016. Annals of surgery.

[33] Fernández-Vigil M, Gil Rodríguez B, Echeverría Trueba J (2020). Fire safety strategies to reduce mortality in dwellings occupied by elderly people: the Spanish case. Fire Technol.

[34] Dokter J, Felix M, Krijnen P, Vloemans JF, van Baar ME, Tuinebreijer WE (2015). Mortality and causes of death of Dutch burn patients during the period 2006–2011. Burns.

[35] Li H, Yao Z, Tan J, Zhou J, Li Y, Wu J (2017). Epidemiology and outcome analysis of 6325 burn patients: a five-year retrospective study in a major burn center in Southwest China. Sci Rep.

[36] Othman N, Kendrick D (2010). Epidemiology of burn injuries in the East Mediterranean Region: a systematic review. BMC public health.

[37] Gregg D, Patil S, Singh K, Marano MA, Lee R, Petrone SJ (2018). Clinical outcomes after burns in elderly patients over 70 years: A 17-year retrospective analysis. Burns.

[38] Hosseini S, Rashtchi V, Kamali K, Moghimi M (2017). Epidemiology and outcome of 2,590 burned patients in Northwest Iran. Ann Burns Fire Disasters.

[39] Kausar A, Dayananda R, PS V (2020). An Autopsy Study of Burn Injuries in Elderly Women. Indian J  Forensic Med Toxicol.

[40] Mandell SP, Pham T, Klein MB (2013). Repeat hospitalization and mortality in older adult burn patients. Journal of Burn Care & Research.

